# A Population-Based Study of Healthcare Resource Utilization in Patients with Mitral Valve Prolapse

**DOI:** 10.3390/ijerph17051622

**Published:** 2020-03-03

**Authors:** Sin-Cih Chen, Sudha Xirasagar, Ju-Chi Liu, Yi-Wei Kao, Ben-Chang Shia, Tzong-Hann Yang, Herng-Ching Lin

**Affiliations:** 1 School of Health Care Administration, College of Management, Taipei Medical University, Taipei 110, Taiwan; m911108001@tmu.edu.tw; 2 Department of Health Services Policy and Management, Arnold School of Public Health, University of South Carolina, Columbia, SC 29208, USA; sxirasagar@sc.edu; 3 Department of Internal Medicine, School of Medicine, College of Medicine, Taipei Medical University, Taipei 110, Taiwan; liumdcv@tmu.edu.tw; 4 Research Center of Big Data, College of management, Taipei Medical University, Taipei 110, Taiwan; kyw498762030@gmail.com (Y.-W.K.); stat1001@tmu.edu.tw (B.-C.S.); 5 Graduate Institute of Business Administration, College of Management, Fu Jen Catholic University, New Taipei City 242, Taiwan; 6 Department of Otorhinolaryngology, Taipei City Hospital, Taipei 110, Taiwan; tzonghannyang@gmail.com; 7 Department of Speech, Language and Audiology, National Taipei University of Nursing and Health, Taipei 110, Taiwan; 8 Research Center of Sleep Medicine, College of Medicine, Taipei Medical University, Taipei 110, Taiwan; 9 Sleep Research Center, Taipei Medical University Hospital, Taipei 110, Taiwan

**Keywords:** mitral valve prolapse, utilization, epidemiology, big data

## Abstract

This study investigated differences in the utilization of healthcare services between subjects with mitral valve prolapse (MVP) and comparison subjects using data from Taiwan’s National Health Insurance population-based database, 138,493 patients with MVP (study group) and 138,493 matched patients without MVP (comparison group). We calculated the utilization of healthcare services in the year 2016 for each study sample. Patients with MVP had more outpatient cardiological services during the year (5.3 vs. 0.7, *p* < 0.001) and higher outpatient cardiology costs (US$226.0 vs. US$30.8, *p* < 0.001) than patients without MVP. As expected, patients with MVP had a longer inpatient stay (0.5 vs. 0.1, *p* < 0.001) and higher inpatients costs (US$158.0 vs. US$22.9, *p* < 0.001) than patients without MVP for cardiology services. Furthermore, patients with MVP also had more outpatient non-cardiology services (20.8 vs. 16.5, *p* < 0.001) and associated costs (US$708.3 vs. US$518.7, *p* < 0.001) than patients without MVP in the year 2016. Multiple regression analysis indicated that patients with MVP had higher total costs for all healthcare services than patients without MVP after adjusting for the urbanization level, monthly income, and geographic region. This study demonstrated that healthcare utilization by patients with MVP is substantially higher than comparison patients. Future studies are encouraged to explore MVP treatment with less expensive modalities while maintaining care quality and without jeopardizing patient outcomes.

## 1. Introduction

Mitral valve prolapse (MVP) is defined as abnormal bulging of the mitral valve leaflets, which is a risk factor for mitral regurgitation during ventricular systole. The prevalence of MVP is 2–3%, which is a common heart condition. Based on prevalence estimates, MVP affects an estimated 176 million people worldwide [1,2]. Many studies have reported that MVP is more common among females, one example study showing 64% of MVP patients being female [3]. Other studies showed MVP that is found as a comorbidity in association with many cardiovascular conditions including heart failure, infective endocarditis, mitral regurgitation (MR), and transient ischemic attacks [4].

Previous research has shown that MVP and the related co-morbidities and complications impact patients’ quality of life and lead to increased healthcare utilization [1,5,6]. Two studies indicated that the annual costs of heart failure were €2.8 billion in Germany and $392 billion in America [5,7]. In the USA Vassileva et al. showed that the mean cost of mitral valve repair and replacement were respectively $30,720 and $45,485 [8], with total nation-wide costs of heart-valve disease amounting to $23.4 billion including $7.6 billion accounted for by mitral valve disease [9]. Costs are also increasing with time: the cost of hospitalization for a patient with aortic valve disease increased from $31,909 in 2000 to $38,172 in 2012 [5,10].

Although numerous studies have reported on the medical care cost of MVP, the excess costs of MVP patients over patients without MVP is not documented. This study investigated the differences in healthcare utilization between subjects with and without MVP using data from Taiwan’s National Health Insurance population-based database.

## 2. Methods

### 2.1. Database

Patients were identified from the Taiwan National Health Insurance Research Dataset, which is made available to scientists in Taiwan for research purposes. The NHIRD includes all medical claims data and registry files for about 22.6 million enrollees, representing about 98% of the Taiwanese population. The NHIRD is one of the largest and most comprehensive population-based datasets available in the world, allowing researchers to track medical utilization for all enrollees since the beginning of the Taiwan National Health Insurance (NHI) program in 1995.

The study was approved by the Institutional Review Board of Taipei Medical University (TMU-JIRB N201802051).

### 2.2. Study Sample

This cross-sectional study included a study group and comparison group. The study group was selected by first identifying 230,983 patients who had ever visited ambulatory care centers with a diagnosis of MVP (ICD-9-CM code 424.0) between 1 January 2014 and 31 December 2015. Administrative datasets have always been suspected of low diagnosis validity. To improve validity, we included 151,557 patients who had at least two office visit/hospitalization episodes with a diagnosis of MVP. We excluded 3228 patients aged <18 years, as well as 6889 patients who died in 2016 to ensure 1-year follow-up for study patients, resulting in a study group sample of 138,493 patients with MVP.

To identify patients for the comparison group, we first excluded all patients who had ever had a history of MVP since initiation of the NHI program in 1995. We then calculated a propensity score for all the remaining enrollees in the NHIRD using available patient demographic variables, age, sex, monthly income, geographic location (Northern, Central, Southern and Eastern) and residential urbanization level. Thereafter, we identified one comparison patient per MVP patient by propensity score matching with the MVP patients, defined as those with equal or similar propensity score. The final study sample included 276,986 patients, 138,493 study group patients, and 138,493 comparison group patients.

### 2.3. Variables of Interest

We calculated the utilization of healthcare services in 2016 for each patient using three variables, number of outpatient visits, number of inpatient days, and total costs of outpatient and inpatient treatments. We defined care cost as the patient’s copayment plus aggregate monetary value of itemized costs of all services and disposables provided by medical providers. Furthermore, we classified utilization into cardiology and non-cardiology services. Cardiology services were defined as services provided or overseen by a board-certified cardiologist.

### 2.4. Statistical Analysis

The SAS statistical package (SAS System for Windows, version 9.0, Cary, NC, USA) was used to perform statistical analyses. Descriptive statistical analyses were used to present the frequency, percentage, median and interquartile range of the outcome variables. We used chi-squared tests to explore the significance of differences in income, geographic location, and urbanization between the MVP group and comparison group, and Wilcoxon-Mann-Whitney tests for outcome variables. Multivariable regression analysis was performed to model the logarithm of total costs as a linear function of a set of independent variables. The difference was considered significant if the two-sided *p*-value was ≤0.05.

## 3. Results

The mean age of the sample of 276,986 patients was 54.1 ± 16.8 years. Patients with and without MVP were similar on monthly income, geographic location, and urbanization level (all *p* > 0.999) (Table 1).

Use and costs of healthcare services in 2016 by the two groups is shown in Table 2. As expected, patients with MVP had more outpatient cardiology visits than the comparison group (*p* < 0.001) and higher outpatient cardiological costs (*p* < 0.001; average currency exchange rate in 2011 was US$1.00 ≈ New Taiwan Dollar (NT$) 29). Also as expected, patients with MVP had a more days of inpatient stay for cardiology services (*p* < 0.001) and higher inpatient cardiology care costs (*p* < 0.001).

Patients with MVP also had more outpatient non-cardiology services than the comparison group (*p* < 0.001) and higher outpatient non-cardiological costs (*p* < 0.001). Similarly, patients with MVP had slightly more non-cardiology inpatient days (*p* < 0.001) and higher inpatient costs (*p* < 0.001).

Regarding use and costs of all healthcare services, patients with MVP had more outpatient visits than comparison patients (*p* < 0.001), higher outpatient costs (*p* < 0.001), more inpatient days (*p* < 0.001), higher inpatient costs (*p* < 0.001), and total healthcare costs (*p* < 0.001).

Table 3 presents the results of multiple regression analyses for adjusted relationships between log-transformed total health care cost in the year and MVP status. Patients with MVP had higher total health care costs than patients without MVP after adjusting for the urbanization level, monthly income, and geographic region.

## 4. Discussion

To our knowledge, this population-based study is the first to compare differences in healthcare utilization between patients with and without MVP. Patients with MVP used significantly more cardiology services, both outpatient and inpatient care. MVP patients also used outpatient non-cardiology services more and had higher costs than comparison patients with similar findings for total inpatient days and inpatient care costs. After adjusting for urbanization, monthly income, and geographic region, the total healthcare costs for MVP patients were higher than those for comparison patients.

Although the study does not establish a causal association between MVP and high healthcare utilization, certain plausible interpretations exist. As reported by previous studies, patients with MVP often have comorbid conditions or complications such as heart failure, infective endocarditis, mitral regurgitation, and transient ischemic attacks [4]. Additionally, the medical care costs of these co-morbidities and complications are significantly high. The annual medical care cost of heart failure in the USA ($39.2 billion) was approximately 2% of the total healthcare budget. With mitral valve disease, the surgical group incurred a total cost of valve repair and replacement of €80.6 million, and in non-surgical group is €163.9 million [5].

Two potential reasons may explain the differences in non-cardiology services between MVP and comparison groups. First, primary MVP may be associated with other non-cardiology conditions such as thoracic skeletal abnormalities, von Willebrand syndrome, and hypomastia [11,12,13,14], which contribute to increased use of non-cardiology services. MVP secondary to a connective tissue disorder, including Marfan syndrome, MASS phenotype, Ehlers–Danlos syndrome, and osteogenesis imperfecta, etc. is also associated with utilization related to the primary condition [15]. One study showed that heritable connective tissue disorders were associated with aortic regurgitation (0.6%, adjusted OR: 6.40, 95%CI 4.74 to 8.66) and mitral regurgitation [16]. In particular, patients with these disorders may develop non-cardiology conditions, including ocular abnormalities, dural ectasia and pulmonary disease [17] which increase non-cardiology care costs. In addition, there was a study mentioned that Marfan syndrome mainly impacts ocular, skeletal, and cardiovascular systems. Ocular findings include risk for myopia (the most common), ectopia lentis (individuals affected by approximately 60%), retinal detachment, glaucoma, and early cataract formation. Additionally, bone overgrowth and joint laxity, scoliosis, pectus excavatum, and pectus carinatum are about skeletal system findings. In brief, utilization of cardiological and non-cardiological services among patients with MVP may be raised by these potential factors.

The primary contribution of this study is being based on large-scale population-based data from the single-payer health system of Taiwan, providing a large sample size with adequate statistical power, and minimize selection bias in both the study group and comparison groups. Some study limitations are noted however. First, no data is available on behavioral factors such as diet, habits and frequency of exercise, alcohol consumption, and educational level. Second, the database has no data on severity of MVP. Further, indirect costs associated with MVP such as missed work days are not estimated. Third, this study did not account for medical conditions in studying the association of MVP with healthcare utilization. It is possible that part of the observed higher care utilization by MVP patients could be due to higher prevalence of chronic diseases such as diabetes, hypertension, and dyslipidemia, as well as other comorbid conditions such as heart failure, infective endocarditis, mitral regurgitation, and transient ischemic attacks. More studies are needed to identify the magnitude of increased healthcare utilization that is attributable to MVP. Finally, we were unable to account for diagnosis coding errors nor for healthcare utilization bias. This could manifest in the form of differential propensity for MVP and comparison patients to seek healthcare services, or undiagnosed MVP among comparison patients who may not have sought care that results in an MVP diagnosis. The diagnosis of MVP was identified using ICD-9-CM codes reported by physicians and hospitals.

Despite these limitations, this study demonstrates that healthcare utilization by MVP patients is substantially higher than comparison subjects. Other population-based epidemiological studies are required to confirm the observed findings in other regions or countries. Additional studies to explore treatment of MVP patients with less expensive modalities while maintaining the quality of medical care may be warranted to reduce unnecessary healthcare costs.

## Figures and Tables

**Table 1:** Demographic characteristics of patients with mitral valve prolapse (MVP) and comparison patients (*n* = 276,986).

Variable	Patients with MVP (*n* = 138,493)		Comparison Patients (*n* = 138,493)		*p* Value
	Total no.	Percent (%)	Total no.	Percent (%)	
Males	49,686	35.9	49,685	35.9	0.997
Age, mean (SD)	54.1 (16.8)		54.1 (16.8)		0.998
Urbanization level					>0.999
1 (most urbanized)	46,586	33.6	46,586	33.6	
2	29,358	21.2	29,360	21.2	
3	9680	7.0	9679	7.0	
4	12,044	8.7	12,045	8.7	
5 (least urbanized)	40,825	29.5	40,823	29.5	
Monthly income (US$)					>0.999
$1~530	34,204	24.7	34,206	24.7	
$530~830	53,394	38.6	53,394	38.6	
≥$830	50,895	36.7	50,893	36.7	
Geographic region					>0.999
Northern	85,417	61.7	85,418	61.7	
Central	29,426	21.2	29,425	21.2	
Southern	22,225	16.0	22,224	16.0	
Eastern	1425	1.0	1426	1.0	

**Table 2:** One-year Healthcare use and costs (US$)—Patients with and without mitral valve prolapse (MVP) and comparison subjects.

Variable	Patients with MVP (*n* = 138,493)		Comparison Patients (*n* = 138,493)		*p* Value
	Median	[Q1, Q3]	Median	[Q1, Q3]	
Cardiological services					
Outpatients services (no. of visits)	3	[1, 9]	0	[0, 0]	<0.001
Outpatient costs (US$)	154	[17, 320]	0	[0, 0]	<0.001
Inpatient days	0	[0, 0]	0	[0, 0]	<0.001
Inpatient costs (US$)	0	[0, 0]	0	[0, 0]	<0.001
Total costs (US$)	158	[20, 326]	0	[0, 0]	<0.001
Non-cardiological services					
Outpatients services (no. of visits)	16	[7, 29]	12.0	[6, 22]	<0.001
Outpatient costs (US$)	310	[116, 707]	201	[77, 489]	<0.001
Inpatient days	0	[0, 0]	0	[0, 0]	<0.001
Inpatient costs (US$)	0	[0, 0]	0	[0, 00]	<0.001
Total costs (US$)	366	[136, 913]	227	[86, 601]	<0.001
All health services					
Outpatients services (no. of visits)	21	[12, 35]	13.0	[6, 23]	<0.001
Outpatient costs (US$)	542	[274, 1005]	218. 5	[82, 537]	<0.001
Inpatient days	0	[0, 0]	0	[0, 0]	<0.001
Inpatient costs (US$)	0	[0, 0]	0	[0, 0]	<0.001
Total costs (US$)	605	[295, 1278]	246	[92, 655]	<0.001

**Table 3:** Multiple regression analysis results showing adjusted relationships between log-cost of all healthcare services and mitral valve prolapse (MVP).

Variable	Log (All Health Services Costs)	
	B	*p* Value
Group		
Subjects with MVP	0.929	<0.001
Comparison subjects	Reference	
Urbanization level		
1 (most urbanized)	−0.072	<0.001
2	−0.050	<0.001
3	−0.057	<0.001
4	0.071	<0.001
5 (least urbanized)	Reference	
Monthly income (US$)		
$1~530	0.248	<0.001
$530~830	Reference	
≥$830	−0.102	<0.001
Geographic region		
Northern	Reference	
Central	0.133	<0.001
Southern	0.130	<0.001
Eastern	0.190	<0.001
